# Developing Clostridia as Cell Factories for Short- and Medium-Chain Ester Production

**DOI:** 10.3389/fbioe.2021.661694

**Published:** 2021-06-07

**Authors:** Qingzhuo Wang, Naief H. Al Makishah, Qi Li, Yanan Li, Wenzheng Liu, Xiaoman Sun, Zhiqiang Wen, Sheng Yang

**Affiliations:** ^1^School of Food Science and Pharmaceutical Engineering, Nanjing Normal University, Nanjing, China; ^2^Department of Environmental Sciences, Faculty of Meteorology, Environment and Arid Land Agriculture, King Abdulaziz University, Jeddah, Saudi Arabia; ^3^College of Life Sciences, Sichuan Normal University, Chengdu, China; ^4^Huzhou Center of Industrial Biotechnology, Shanghai Institutes of Biological Sciences, Chinese Academy of Sciences, Shanghai, China; ^5^Key Laboratory of Synthetic Biology, CAS Center for Excellence in Molecular Plant Sciences, Shanghai Institute of Plant Physiology and Ecology, Chinese Academy of Sciences, Shanghai, China

**Keywords:** *Clostridium*, ester, lipase, alcohol acyltransferase, synthetic biology

## Abstract

Short- and medium-chain volatile esters with flavors and fruity fragrances, such as ethyl acetate, butyl acetate, and butyl butyrate, are usually value-added in brewing, food, and pharmacy. The esters can be naturally produced by some microorganisms. As ester-forming reactions are increasingly deeply understood, it is possible to produce esters in non-natural but more potential hosts. Clostridia are a group of important industrial microorganisms since they can produce a variety of volatile organic acids and alcohols with high titers, especially butanol and butyric acid through the CoA-dependent carbon chain elongation pathway. This implies sufficient supplies of acyl-CoA, organic acids, and alcohols in cells, which are precursors for ester production. Besides, some Clostridia could utilize lignocellulosic biomass, industrial off-gas, or crude glycerol to produce other branched or straight-chain alcohols and acids. Therefore, Clostridia offer great potential to be engineered to produce short- and medium-chain volatile esters. In the review, the efforts to produce esters from Clostridia via *in vitro* lipase-mediated catalysis and *in vivo* alcohol acyltransferase (AAT)-mediated reaction are comprehensively revisited. Besides, the advantageous characteristics of several Clostridia and clostridial consortia for bio-ester production and the driving force of synthetic biology to clostridial chassis development are also discussed. It is believed that synthetic biotechnology should enable the future development of more effective Clostridia for ester production.

## Introduction

Short- and medium-chain volatile esters (C2–C12) with flavors and fruity fragrances are usually value-added in brewing, food, and pharmacy (Rodriguez et al., 2014; Aleksander et al., 2019). For example, ethyl acetate, ethyl lactate, butyl acetate, and ethyl hexanoate are the main components of the flavor substances in Baijiu (Chinese liquor) (Yi et al., 2019). With the expansion of the application field, the demand of esters continues to rise in recent years.

Traditionally, short- and medium-chain fatty acid esters are mainly produced by concentrated sulfuric acid-mediated esterification of acids and alcohols (Cull et al., 2003). This method has certain risks in terms of safety, health, and environment, because it usually causes serious equipment corrosion, as well as a large amount of wastewater and residues (Jermy and Pandurangan, 2005). The recently developed ionic liquid catalytic method can alleviate these problems to some extent, but it is expensive and not stable (Tankov et al., 2017). Compared to chemical methods, biosynthesis via enzyme catalysis or metabolic engineering is much more environmentally friendly and is expected to be an alternative. Indeed, the esters can be naturally produced by some yeasts and lactic acid bacteria, but the efficiency is far from satisfactory (Mukdsi et al., 2009; Kruis et al., 2018a). Therefore, a lot of efforts have been paid to develop non-natural but more potential strains as microbial cell factories for short- and medium-chain volatile ester production (Rodriguez et al., 2014; Kruis et al., 2017).

Clostridia are especially suitable hosts for ester production due to the diversity of abundant precursors, substrates, and products (Moon et al., 2016; Noh et al., 2019). In the review, we summarized the advances of ester production by Clostridia including *in vitro* lipase catalysis and *in vivo* acyltransferase reaction. Besides, we suggested some promising clostridial chassis for bio-ester production and discussed the driving force of synthetic biology in this field.

## Enzymes and Pathways in Microorganisms for Ester Production

There are mainly four kinds of ester-forming reactions reported in microorganisms that naturally produce esters. Correspondingly, four kinds of ester synthases including esterase (lipase), hemiacetal dehydrogenase, Baeyer–Villiger monooxygenases, and alcohol acyltransferase (AAT) are involved (Aleksander et al., 2019). Among them, esterase and AAT-mediated reactions are often used for ester overproduction (Aleksander et al., 2019; Noh et al., 2019).

It is a classic strategy to adapt lipase to catalyze the esterification reaction of organic acids and short-chain alcohols (Stergiou et al., 2013; Aleksander et al., 2019). Unfortunately, for the *in vitro* catalytic system, enzymes and substrates need to be produced dedicatedly, which caused complicated process routes and huge equipment costs in most cases (Stergiou et al., 2013; Aleksander et al., 2019).

Theoretically, the integration of enzyme and substrate production and catalytic reaction in one reactor is expected to dramatically reduce costs. Therefore, metabolic engineering by the condensation of alcohols and acyl-CoA using AATs is an emerging strategy, which enables some microorganisms, such as *Escherichia coli*, *Saccharomyces cerevisiae*, and *Kluyveromyces marxianus*, to produce bio-esters (Rodriguez et al., 2014; Kruis et al., 2017; Löbs et al., 2017; Bohnenkamp et al., 2020). The reported AATs are mainly derived from yeast, including *S. cerevisiae*, *K. marxianus*, *Saccharomyces bayanus*, and *Saccharomyces uvarum* (Fujii et al., 1996; Yoshimoto et al., 1998; Saerens et al., 2006). For example, in *S. cerevisiae*, alcohol acetyltransferases are mainly encoded by the genes *ATF1*, *ATF2*, *EEB1*, *EHT1*, and *EAT1* (Fujii et al., 1996; Lilly et al., 2006; Saerens et al., 2006; Kruis et al., 2018a,b). These genes have a certain compensatory effect on each other, but the catalytic activity and substrate preference of these AATs are not completely the same. Besides, AATs from different strain sources usually exhibited different catalytic activities and substrate selectivity, which explains why *E. coli* produces multiple esters after different (and even the same) AATs are introduced (Rodriguez et al., 2014).

In addition to the thoughtful selection and refined expression of AATs, challenges to balance the metabolic pathways for rational precursor distribution need to be addressed. This is because acyl-CoA is an indispensable precursor of esters, as well as fatty acids and alcohols. The metabolism of esters, fatty acids, and fatty alcohols inevitably competes for acyl-CoA (Aleksander et al., 2019; Noh et al., 2019). In *E. coli*, *S. cerevisiae* and *K. marxianus*, acyl-CoA is usually of tight supply, and the metabolic and regulatory networks for acyl-CoA synthesis and consumption are rather complicated. It challenges in distributing precursors reasonably (Rodriguez et al., 2014; Kruis et al., 2017; Löbs et al., 2017).

**FIGURE 1:**
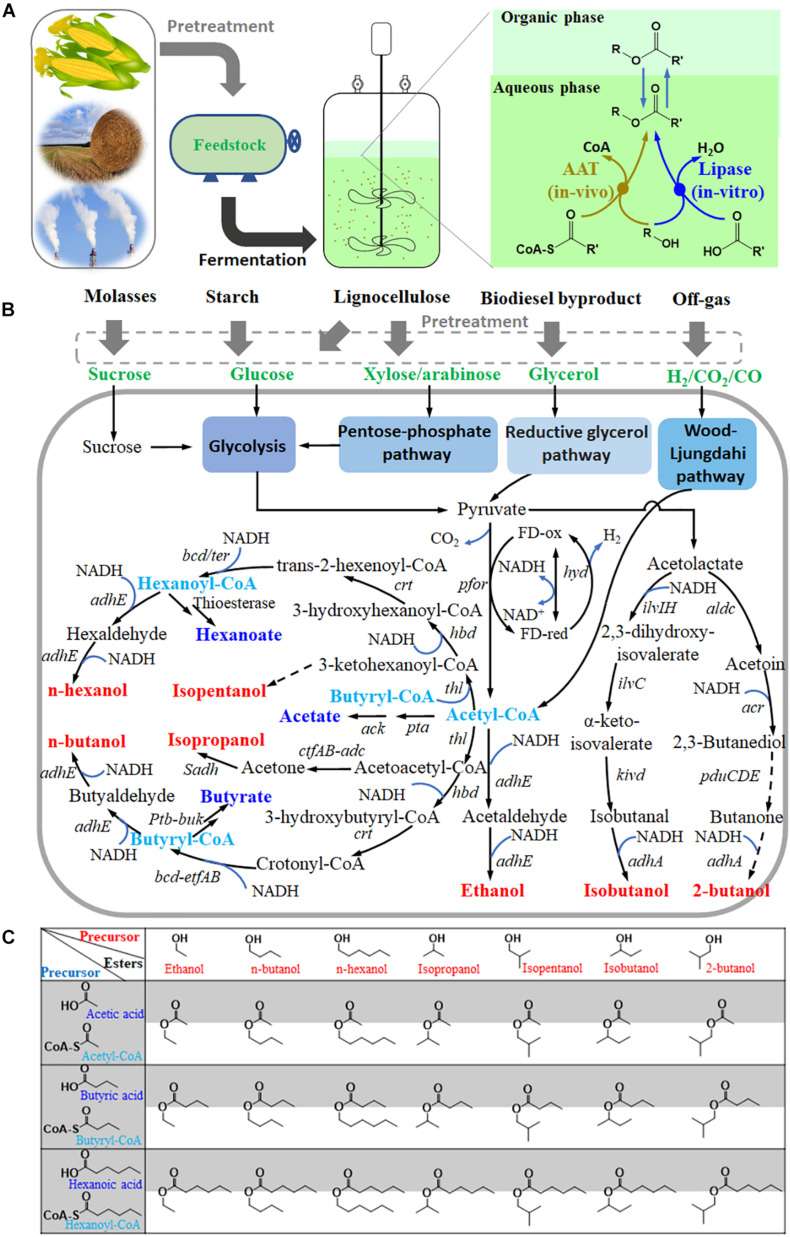
Clostridia are promising cell factories for short- and medium-chain ester production. **(A)**, process and methods for ester production by Clostridia from various feedstocks; **(B)**, metabolic pathway for ester precursors (short-chain acids or acyl CoA, and alcohols) in some Clostridia; **(C)**, matrix of esters putatively produced by Clostridia based on the above precursors. *pfor*, pyruvate: ferredoxin oxidoreductase; *thl*, acetyl-CoA acetyltransferase; *hbd*, beta-hydroxybutyryl-CoA dehydrogenase; *crt*, crotonase; *bcd/ter*, butyryl-CoA dehydrogenase; *adhE* or *adhE1/2*, acetaldehyde/ethanol dehydrogenase; *pta*, phosphotransacetylase; *ack*, acetate kinase; *ctfAB*, acetate/butyrate-acetoacetate COA-transferase; *adc*, acetoacetate decarboxylase; *Sadh*, primary/secondary alcohol dehydrogenase; *ptb*, phosphate butyryl-transferase; *buk*, butyrate kinase; *hyd*, ferredoxin hydrogenase; *bdhA/B*, butanol dehydrogenase; *edh*, alcohol dehydrogenase; *ilvIH*, acetolactate synthase; *ilvC*, keto-acid reductoisomerase; *kivd*, α-ketoisovalerate decarboxylase; *adhA*, alcohol dehydrogenase; *aldc*, acetolactate decarboxylase; acr, acetoin reductase; *pduCDE*, diol dehydratase. Dash lines indicate putative pathways.

In contrast, Clostridia can produce a variety of volatile organic acids and alcohols (Figure 1), especially butanol and butyrate through the CoA-dependent carbon chain elongation pathway (Tracy et al., 2012; Cho et al., 2015). This implies sufficient supplies of acyl-CoA, organic acids, and alcohols in cells, which are precursors for ester production. Therefore, Clostridia offer great potential to be engineered to produce short- and medium-chain volatile esters. Recently, many important progresses, including lipase catalysis of fermentation broth, AAT heterologous expression, and exploration of different clostridial chassis, have been made in Clostridia-assisted ester production.

## Lipases Mediated Esterification of Alcohols and Acid From Clostridia

*Clostridium* is a very important category of prokaryotes, composed of nearly 200 different species (Wiegel et al., 2006). Some non-pathogenic species such as *Clostridium acetobutylicum* and *Clostridium beijerinckii* are known as solventogenic Clostridia because they can utilize starch, molasses, and other sugars to produce bulk chemicals such as ethanol, butanol, and acetone (Lee et al., 2008). Some other species can directly use lignocellulose, glycerol, or syngas (H_2_/CO_2_, CO) as a sole carbon source to produce short-chain organic acids or alcohols (Ren et al., 2016).

These alcohols and acids are natural substrates for lipase-catalyzed esterification reactions. Therefore, adding lipase to the clostridial fermentation broth is a common strategy for ester production (Noh et al., 2019). In 2013, van den Berg et al. (2013) realized butyl butyrate biosynthesis, by adding commercially available *Candida antarctica* lipase B (CaLB; Novozym 435) into the fermentation broth of *C. acetobutylicum*. Meanwhile, hexadecane was adapted to extract butyl butyrate to the organic phase, which helped in product separation and reduced the toxicity of butyl butyrate. Unfortunately, the butyl butyrate titer is only 4.9 g/L in the extractant phase because of low butyrate accumulation during ABE fermentation. Therefore, butyrate was supplemented in a later study in *Clostridium* sp. strain BOH3 broth (Xin et al., 2016). As for the fermentation broth of *Clostridium tyrobutyricum*, a butyrate hyper-producing strain, butanol supplementation is necessary for butyl butyrate production (Zhang et al., 2017).

In order to reduce substrate costs and fermentation process complexity caused by butyrate or butanol addition, a clostridial consortium composed of *C. tyrobutyricum* and *C. beijerinckii* was established (Cui et al., 2020a). In the consortium, butyrate produced by *C. tyrobutyricum* and butanol and isopropanol produced by *C. beijerinckii* are catalyzed by exogenous lipase to form butyl butyrate and isopropyl butyrate, respectively. In another study (Seo et al., 2017), a *C. beijerinckii spo0A* (a critical regulator to shift metabolism from acidogenesis to solventogenesis) mutant was used for ester production. Since transition from butyrate to butanol is disrupted in the strain, it could produce more butyrate but less butanol compared with the wild type. Accordingly, the addition of butyrate and butanol is avoided, but exogenous lipase is still indispensable.

Although it has been reported that indigenous lipases of *Clostridium* sp. strain BOH3 can be induced by olive oil or Bio-OSR (Xin et al., 2016), indigenous lipases (with low expression level and enzymatic activity) are often not enough, and additional lipases are still required to further increase butyl-butyrate production. What is more, lipase source, lipase loading dosage, and other factors like extractant, agitation speed, and pH also affected the performance of lipase-mediated esterification in fermentation broth (Xin et al., 2016; Zhang et al., 2017).

The bottleneck of lipase-mediated esterification lies in the cost of exogenously added lipase. Fortunately, immobilized lipase and optimal reaction conditions may make the cost acceptable. An alternative option is overexpressing and secreting heterologous lipases by *Clostridium*. However, there has been almost no progress in lipase overexpression in *Clostridium* until now, because it is really challenging to engineer *Clostridium* to secrete proteins well (Wen et al., 2020c). By comparison, the *in vivo* AATs-dependent pathway is more thermodynamically favorable in an aqueous fermentation environment, but more dependent on hosts (Noh et al., 2019).

## Alcohol Acyltransferase-Mediated Ester Synthesis in Clostridia

Ester production in Clostridia by condensation of alcohols and acyl-CoA can be traced back to 2006 (Horton and Bennett, 2006). Horton and Bennett (2006) successfully overexpressed the *ATF2* gene from *S. cerevisiae* in wild *C. acetobutylicum* and a mutant strain M5, respectively, and realized butyl acetate production in strain M5 from glucose for the first time. Noh et al. also observed bio-ester synthesis in *C. acetobutylicum* after introducing alcohol acyltransferases (AATs) from *Fragaria x ananassa* (strawberry) or *Malus* sp. (apple), respectively (Noh et al., 2018). Interestingly, butyl butyrate accounted for about 90% of the total esters, while butyl acetate accounted for a very small proportion. In two recent studies, Fang et al. (2020) and Li et al. (2020) expressed ATF1 in *C. beijerinckii* and *Clostridium diolis*, respectively; the generated strains produced 5.42 and 1.37 g/L of butyl acetate as the main ester products from glucose. The type difference in the main ester products in the above studies may be attributed to substrate selectivity of AATs from different sources (Noh et al., 2018; Aleksander et al., 2019).

In addition to mesophilic Clostridia, the thermophilic Clostridia are also promising hosts for bio-ester production, because higher temperature could facilitate the downstream ester separation (Mazzoli and Olson, 2020). Seo et al. (2019) demonstrated that a thermostable chloramphenicol acetyltransferase from *Staphylococcus aureus* (CATSa) can work as a potential AAT in *Clostridium thermocellum*. CATSa heterologous expression in *C. thermocellum* has enabled the production of ethyl acetate and isobutyl acetate directly from cellulose. A potential drawback is ester degradation caused by the endogenous carbohydrate esterases (CEs), which hindered ester accumulation *in vivo*. Therefore, in a subsequent study, Seo et al. identified and disrupted two putative CEs (encoded by *Clo1313_0613* and *Clo1313_0693*) in *C. thermocellum*, which alleviated ester degradation and further improved isobutyl acetate production by almost 10-fold (Seo et al., 2020).

Generally, Clostridia have been proven to be potential cell factories for ester production. However, these studies mainly focus on the construction and optimization of the ester production process in Clostridia. The characteristics and advantages of Clostridia have not been fully utilized.

## Several Potential Clostridial Hosts for Ester Synthesis

Clostridial hosts for ester synthesis have been expanded from typical solventogenic Clostridia to some unconventional strains. These Clostridia with advantageous characteristics in substrate utilization and ester precursor accumulation are worth exploring and developing as novel ester production chassis.

*Clostridium tyrobutyricum* is one of the most efficient butyrate-producing Clostridia, which can produce about 50 g/L butyrate in batch fermentation under optimized culture conditions (Fu et al., 2017; Bao et al., 2020). Different from the solventogenic Clostridia such as *C. acetobutylicum* and *C. beijerinckii*, there is a special acetate and butyrate reassimilation mechanism in the strain. With its unique CoA transferase CAT1, acetate and butyrate can be efficiently reconverted into acetyl-CoA and butyryl-CoA, respectively, without coupling with acetone synthesis (Bao et al., 2020). The introduction of an aldehyde/alcohol dehydrogenase (encoded by *adhE2*) from *C. acetobutylicum* enabled more than 10 g/L butanol produced from glucose (Yu et al., 2011). Recently, Zhang et al. (2018) developed a gene-editing tool applicable in *C. tyrobutyricum* based on its endogenous Type IB CRISPR/Cas system. They found that when the *cat1* gene was replaced in-frame by *adhE2*, the butanol titer of the mutant reached an unprecedented 26.2 g/L. Moreover, the final concentration of by-products acetate and butyrate reached 15.2 and 2.4 g/L, respectively. High concentrations of butanol, acetate, and butyrate implied the sufficient supply of ester precursors, which may contribute to achieve a very high titer of butyl acetate or butyl butyrate.

Similar to *C. tyrobutyricum*, the main product of *Clostridium cellulovorans* is also butyrate, while the difference is that it can directly grow on lignocellulosic biomass (Sleat et al., 1984). Interestingly, this strain harbors a complete CoA-dependent butanol synthesis pathway without coupled acetone production according to the prediction of KEGG, but it can hardly produce butanol. Modular metabolic engineering has enabled the strain to produce 4.96 g/L butanol from alkali-extracted corn cob (AECC) in 120 h, with 4.81 g/L butyrate and 4.14 g/L acetate residual in broth (Wen et al., 2020b), suggesting sufficient precursors for the synthesis of butyl acetate or butyl butyrate. Recently, Fang et al. claimed that they had realized butyl acetate production in *C. cellulovorans* by overexpressing *ATF1* and *adhE1*, but the detailed experimental data was not shown (Fang et al., 2020). Like *C. cellulovorans*, *Clostridium cellulolyticum* and *C. thermocellum* are also important cellulolytic Clostridia, and *C. thermocellum* has been proven to produce isobutyl acetate and isobutyl isobutyrate directly from cellulose (Seo et al., 2019; Zhang J. et al., 2020). One putative obstacle is that there are no complete pathways from acetyl-CoA to butyrate and butanol existing in *C. cellulolyticum* and *C. thermocellum*. Encouragingly, they have been successfully engineered to produce butanol (Gaida et al., 2016; Tian et al., 2019). In general, cellulolytic Clostridia offered a chance to produce esters from lignocellulose by consolidated bioprocessing.

Another reason why *C. cellulovorans* has potential as an excellent candidate host is its CO_2_ fixation ability (Shinohara et al., 2013), although its fixation efficiency is much lower than gas-fermenting Clostridia. Gas-fermenting Clostridia is a major type of chemoautotrophic carbon-fixing bacteria, in which *Clostridium ljungdahlii*, *Clostridium autoethanogenum*, *Clostridium carboxidivorans* have been adapted for ethanol and butanol production from industrial waste gas (Latif et al., 2014; Bengelsdorf and Durre, 2017). Gas-fermenting Clostridia uptake and fix CO_2_, CO, and H_2_ by a Wood–Ljungdahl (WL) pathway (Fast et al., 2015). The energy metabolism and product synthesis in these strains may be completely different under different growth conditions, which result in product diversity (Bengelsdorf and Durre, 2017). Apart from acetate and ethanol, butyrate can be detected in the fermentation broth of *C. carboxidivorans* (Fernandez-Naveira et al., 2017a), *Clostridium drakei* (Gossner et al., 2008), *Clostridium magnum* (Groher and Weuster-Botz, 2016), and *Clostridium scatologenes* (Song et al., 2014). Moreover, butanol, hexanoate, and hexanol can also be produced by *C. carboxidivorans* (Fernandez-Naveira et al., 2017a) and *C. drakei* (Fernandez-Naveira et al., 2017b). The special substrate spectrum and product (or precursor) diversity make gas-fermenting Clostridia very suitable for different ester synthesis.

## Artificial Clostridial Consortia Offer Special Advantages for Bio-Ester Synthesis

Ester synthesis is a complex process involving multiple steps, such as the utilization of substrates, precursor production, lipase or AAT expression, and catalysis (Aleksander et al., 2019). A mixed-culture strategy has proven successful in complex biological processes (Cui et al., 2020b; Wen et al., 2020c). The members in consortia can take on different tasks and exert their unique advantages, thereby reducing the burden, expanding the spectrum of substrates, increasing product diversity, and improving the efficiency of ester synthesis. In an aforementioned study, the titer of butyl butyrate produced from coculture of *C. beijerinckii* (the butanol producer) and *C. tyrobutyricum* (the butyrate producer) is about 10-fold obtained from the *C. beijerinckii* monoculture, implying great potential (Cui et al., 2020a). The consortia that have been adapted for cellulosic butanol production [for example, cellulolytic *C. cellulovorans* and solventogenic *C. beijerinckii* (Wen et al., 2017)] and syngas fermentation [for example, gas-fermenting *C. ljungdahlii* and hexanoate-producing *Clostridium kluyveri* (Richter et al., 2016)] could be engineered for bio-ester production by simply introducing AATs or adding lipase.

Other Clostridia that have the potential to serve as production hosts for bio-esters but have not been discussed are summarized in Table 1. According to the table, these distinctive Clostridia and clostridial consortia endow new possibilities for bio-ester synthesis in the aspects of efficiency improvements, broad substrate spectrum, and product diversity. However, the above studies rarely involve the complicated modification of the clostridial host, including metabolic pathway reconstruction, stress resistance modification, and refined expression regulation of AATs, which implies great potential of clostridial synthetic biotechnology to improve bio-ester production.

**TABLE 1:** Potential Clostridia for the production of short- and medium-chain esters.

**Strains or consortia**	**Genetic tools**	**Substrates**	**Acid or acyl-CoA precursors**		**Alcohol precursors**	**References**
***C. thermocellum*** ^*a*^	Available^*b*^	Lignocellulose, sugars	Acetate	Acetyl-CoA, butyryl-CoA^*c*^, isobutyryl-CoA	Ethanol, **butanol**^*d*^, **isobutanol**	Lin et al., 2015; Seo et al., 2019; Tian et al., 2019; Mazzoli and Olson, 2020; Seo et al., 2020
***C. cellulovorans***	Available	Lignocellulose, sugars	Acetate, butyrate	Acetyl-CoA, butyryl-CoA	Ethanol, **butanol**	Sleat et al., 1984; Yang et al., 2015; Wen et al., 2020b
*C. cellulolyticum*	Available	Lignocellulose, sugars	Acetate	Acetyl-CoA, butyryl-CoA	Ethanol, **butanol**, **isobutanol**	Higashide et al., 2011; Gaida et al., 2016
*C. phytofermentans*	Unavailable	Lignocellulose, sugars	Acetate	Acetyl-CoA	Ethanol	Tolonen et al., 2015
*C. clariflavum*	Unavailable	Lignocellulose, sugars	Acetate	Acetyl-CoA	Ethanol	Artzi et al., 2015
*C. termitidis* CT111	Unavailable	Lignocellulose, sugars	Acetate	Acetyl-CoA	Ethanol	Munir et al., 2016
***C. acetobutylicum***	Available	Starch, sugars, glycerol	Acetate, butyrate	Acetyl-CoA, butyryl-CoA	Ethanol, butanol, **2,3-butanediol, 1,3-propanediol**	van den Berg et al., 2013; Cho et al., 2015; Noh et al., 2019
***C. beijerinckii***	Available	Starch, sugars, glycerol	Acetate, butyrate	Acetyl-CoA, butyryl-CoA	Ethanol, butanol	Seo et al., 2017; Fang et al., 2020
***C. tyrobutyricum***	Available	Starch, sugars, glycerol	Acetate, butyrate	Acetyl-CoA, butyryl-CoA	Ethanol, **butanol**	Yu et al., 2011; Fu et al., 2017; Zhang et al., 2018
*C. saccharoperbutylacetonicum*	Available	Starch, sugars, glycerol	Acetate, butyrate	Acetyl-CoA, butyryl-CoA	Ethanol, butanol	Noguchi et al., 2013
*C. kluyveri*	Available	Starch, sugars, glycerol	Acetate, butyrate, hexanoate, octanoate	Acetyl-CoA, butyryl-CoA, hexanoyl-CoA, octanoyl-CoA	Ethanol, butanol, hexanol	Seedorf et al., 2008; Steinbusch et al., 2011
*C. propionicum*	Available	Starch, sugars, glycerol	Acetate, propionate lactate, succinate	Acetyl-CoA	Propanol	Johns, 1952; Barbirato et al., 1997
*C. pasteurianum*	Available	Starch, sugars, glycerol	Acetate, butyrate	Acetyl-CoA, butyryl-CoA	1.3-Propanediol, ethanol, butanol, **1, 2-propanediol**	Malaviya et al., 2012; Groeger et al., 2016; Pyne et al., 2016; Schwarz et al., 2017
***C. diolis***	Available	Starch, sugars, glycerol	Acetate, butyrate	Acetyl-CoA, butyryl-CoA	Ethanol, butanol, 1,3-propanediol	Chen et al., 2018; Li et al., 2020
*C. butyricum*	Available	Starch, sugars, glycerol	Acetate, butyrate, 2-hydroxy-4-methylpentanoate	Acetyl-CoA, butyryl-CoA	Ethanol, 1,3-propanediol	Butel et al., 1995; Chatzifragkou et al., 2011; Miguel Serrano-Bermudez et al., 2017
*Clostridium strain* AK1	Unavailable	L-rhamnose	Acetate, lactate, butyrate	Acetyl-CoA, butyryl-CoA	Ethanol, 1,2-propanediol	Ingvadottir et al., 2018
*C. thermosaccharolyticum* HG-8	Unavailable	Sugars	Acetate, lactate	Acetyl-CoA	Ethanol, 1,2-propanediol	Altaras et al., 2001
*C. thermosaccharolyticum* ATCC 31960	Unavailable	Sugars	Acetate, lactate	Acetyl-CoA	Ethanol, 1,2-propanediol	Cameron and Cooney, 1986
*C. sphenoides*	Unavailable	Sugars	Acetate, lactate	Acetyl-CoA	Ethanol, 1,2-propanediol	Tran-Din and Gottschalk, 1985
*C. ljungdahlii*	Available	H_2_/CO_2_, CO	Acetate, butyrate, lactate	Acetyl-CoA	Ethanol, 2,3-butanediol, **butanol**	Köpke et al., 2010; Zhang L. et al., 2020
*C. carboxidivorans*	Unavailable	H_2_/CO_2_, CO	Acetate, butyrate, lactate	Acetyl-CoA, butyryl-CoA	Ethanol, butanol, hexanol	Shen et al., 2017
*C. autoethanogenum*	Available	H_2_/CO_2_, CO	Acetate, lactate	Acetyl-CoA	Ethanol, 2,3-butanediol, **butanol**	Koepke and Liew, 2012
*C. scatologenes*	Unavailable	H_2_/CO_2_, CO	Acetate, butyrate	Acetyl-CoA, butyryl-CoA	Ethanol	Song et al., 2014
*C. drakei*	Unavailable	H_2_/CO_2_, CO	Acetate, butyrate	Acetyl-CoA, butyryl-CoA	Ethanol	Gossner et al., 2008
*C. thermoaceticum*	Available	H_2_/CO_2_, CO	Acetate	Acetyl-CoA	Ethanol	Pierce et al., 2008
***C. beijerinckii* BGS1 and *C. tyrobutyricum* ATCC 27045**	Available	Starch, sugars	Acetate, butyrate	Acetyl-CoA, butyryl-CoA	Ethanol, butanol	Cui et al., 2020a
*C. thermocellum* and *C. saccharoperbutylacetonicum strain* N1-4	Available	Crystalline cellulose	Acetate, butyrate	Acetyl-CoA, butyryl-CoA	Ethanol, butanol	Shunichi et al., 2011
*C. celevecrescens* N3-2 and *C. acetobutylicum* ATCC 824	Unavailable	Filter paper	Acetate, butyrate	Acetyl-CoA, butyryl-CoA	Ethanol, butanol	Wang et al., 2015
*C. cellulovorans* and *C. beijerinckii*	Available	Alkali extracted corn cobs	Acetate, butyrate	Acetyl-CoA, butyryl-CoA	Ethanol, butanol	Wen et al., 2017; Wen et al., 2020a
*C. thermocellum* and *C. beijerinckii*	Available	Alkali extracted corn cobs	Acetate, butyrate	Acetyl-CoA, butyryl-CoA	Ethanol, butanol	Wen et al., 2014
*C. ljungdahlii and C. kluyveri*	Available	H_2_/CO_2_, CO	Acetate, butyrate, hexanoate	Acetyl-CoA, butyryl-CoA, hexanoyl-CoA	Ethanol	Richter et al., 2016

*^*a*^Bold fonts indicate strains or consortia that have been engineered to enable ester production.*

*^*b*^Double underlines indicate advantages of the strain.*

*^*c*^Single underline indicates limitations of the strain.*

*^*d*^Bold fonts indicate precursors synthesized via genetic modification.*

## Synthetic Biology Will Accelerate Development of Clostridial Ester Cell Factories

Although great progress has been made in bio-ester production, the clostridial potential and advantages have not been fully exploited. Synthetic biology provided many resources and methods for clostridial chassis development, which can be applied to ester production (Joseph et al., 2018; Wen et al., 2020c).

Many genetic manipulation tools such as TargeTron, allelic exchange, CRISPR/Cas system-mediated gene, and base editing tools have been developed in Clostridia (Pyne et al., 2014; McAllister and Sorg, 2019; Wen et al., 2020d). Various genetic operations such as insertion, deletion, substitution, point mutation, and regulation of target gene expression levels can be efficiently implemented in Clostridia, which laid a good foundation for metabolic engineering (Joseph et al., 2018).

Metabolic pathway reconstruction not only increased the titer, yield, and ratio of butanol but also eliminated the by-product acetone in some Clostridia (Cho et al., 2015; Jiang et al., 2015). In addition, it also provides a chance to synthesize some new products (Figure 1), such as long straight-chain alcohols and acids (pentanol, hexanoate, hexanol, octanoate, and octanol) or branched-chain alcohols and acids (1,2-propanediol, isopropanol, isobutanol, 2-butanol, and isopentanol) (Pyne et al., 2016; Ren et al., 2016; Bengelsdorf and Durre, 2017). These products can serve as precursors for novel esters, which may increase the diversity of ester products.

In addition, through metabolic engineering, some Clostridia has been improved in the aspects of hexose/pentose co-fermentation (Gu et al., 2014; Mitchell, 2016), syngas utilization (Bengelsdorf and Durre, 2017; Valgepea et al., 2018), and efficient conversion of glycerol to butanol (Schwarz et al., 2017). These Clostridia can utilize inexpensive and renewable resources such as lignocellulosic biomass, industrial off-gas, or crude glycerol to produce bio-ester, which could further reduce bio-ester cost. However, there are still some putative drawbacks for some clostridial hosts (Moon et al., 2016), such as low recombineering efficiency, complex metabolic regulatory networks, by-products (like acetone) accumulation, and inefficient protein secretion system, which are highly dependent on synthetic biotechnology to solve.

It can be expected that with the further development of synthetic biology, bio-ester production by Clostridia will be closely combined with the novel AAT mining, rational metabolic network simulation and prediction, multi-omics analysis, and artificial consortia design. The ideas and technologies of synthetic biology will accelerate to develop Clostridia as more effective cell factories for ester production.

## Author Contributions

QW, XS, and ZW conceived the project and wrote the manuscript. All authors participated in the discussion, revised the manuscript, and approved the final manuscript.

## Conflict of Interest

The authors declare that the research was conducted in the absence of any commercial or financial relationships that could be construed as a potential conflict of interest.
